# Organic fertilizer application and Mg fertilizer promote banana yield and quality in an Udic Ferralsol

**DOI:** 10.1371/journal.pone.0230593

**Published:** 2020-03-18

**Authors:** Jiangzhou Zhang, Baoshen Li, Junling Zhang, Peter Christie, Xiaolin Li

**Affiliations:** 1 Key Laboratory of Plant-Soil Interactions, Ministry of Education, College of Resources and Environmental Sciences, China Agricultural University, Beijing, China; 2 Guangxi Jinsui Agricultural Group Co., Ltd., Nanning, China; Gifu University, JAPAN

## Abstract

Low soil fertility, high rates of fertilizer application and low yields and quality are major problems in intensive banana production in acid soils of south China. A field experiment was carried out for two years to determine the optimum management practices for maximizing soil health and banana yield and quality. The experiment consisted of an unamended control (CK) and lime (Lime), calcium magnesium phosphate fertilizer (CMP), organic fertilizer (OF), and organic fertilizer combined with calcium magnesium phosphate fertilizer (OFC) treatments. Soil nutrient concentrations and banana shoot biomass, nutrient uptake, yield and fruit quality were determined. Application of lime and CMP was found to increase soil pH and nutrient availability and increase banana yield. Yet, the banana biomass and yields in the Lime and CMP treatments were significantly lower than those in the OF and OFC treatments in which soil organic matter (SOM) content increased. Total soluble solids and soluble sugar contents increased in the CMP and organic fertilizer treatments. A consistent increase in Mg concentrations in banana leaves over the two years in the CMP and organic fertilizer treatments indicates that Mg is essential for banana production and quality. Short-term adding Mg from banana corms increased total soluble solids and soluble sugar content. The application of organic fertilizer combined with CMP or Mg solution is therefore recommended to increase soil health and promote the yield and quality of banana in intensively managed plantations in subtropical regions.

## Introduction

The demand for high-quality food continues to increase with the growing world population and high food quality and quantity are required including fruits such as bananas, peaches and citrus [1,2]. Banana (*Musa* spp.) is an important tropical cash crop that is grown in tropical and subtropical regions. The estimated yield of banana in China averages 31.0 t ha^-1^, a higher value than the world average (20.6 t ha^-1^) in 2016 [3], but lower (32.4 and 42.8 t ha^-1^ yield gap, respectively) than the world average maximum yield (63.4 t ha^-1^). Most importantly, approximately 20–25% of banana fruits are unsuitable for retail sale due to poor quality [4]. Hence, increasing both banana yield and quality is a major challenge and maintenance of high soil quality is a fundamental requirement for optimizing banana production.

Soil quality is the capacity of a specific soil type to function within natural or managed ecosystem boundaries to sustain plant and animal productivity, maintain or enhance water and air quality, and support human health and habitation [5]. Low soil fertility is a common major constraint in banana production [6,7], with banana produced predominantly in Ferralsols or Acrisols in China, Uganda and other parts of the world [8]. The highly weathered soils are characterized by low organic matter content, high acidity, poor soil structure and limited capacity to supply base cations including Ca^2+^ and Mg^2+^ [9,10]. In Uganda the addition of chemical fertilizers has increased soil nutrient availability, foliar nutrient concentrations and banana yields [11]. Farmers in China often apply large amounts of inorganic fertilizers to the soil in order to ensure high banana yields in intensive plantations. For instance, the application rates of N, P and K are 585, 511 and 912 kg ha^-1^, respectively, in Puer city (Yunnan province), southwest China [12]. The application rates of N and P are several times higher than the nutrient requirement of banana plants (448, 59.9 and 1457 kg ha^-1^, respectively) to produce 50 t ha^-1^ [13]. Nutrient imbalance, and in particular the overuse of fertilizer nitrogen, often leads to soil acidification [14], and this accelerates the runoff of base cations such as Ca^2+^ and Mg^2+^ from the soil profile, leading to poor banana quality as these elements are essential for high fruit quality. In contrast, little or no Ca, Mg or micronutrient fertilizers are applied to banana plantations [12]. It is well known that calcium (Ca) makes an important contribution to fruit quality by playing a crucial role in cell wall strength, increasing papaya fruit pulp cell wall thickness and reducing fruit cracking [15,16]. The fruit quality of Ca-deficient banana plants is inferior and the fruit peel splits easily when ripe [17]. Total soluble solids and soluble sugar content are often used to assess banana quality [18]. Potassium (K) plays an important role in photosynthesis and transport of metabolites, hence in banana management K fertilizers are often supplied at sufficient or sometimes excessive rates. By contrast, the importance of Mg is poorly recognized despite the essential function of Mg in photosynthesis and carbohydrate partitioning in plants from source to sink [19]. Large amounts of sugars accumulate in leaves (source) due to impaired phloem transportation to the fruit (sink) under Mg deficiency conditions [20]. Previous studies show that Mg concentrations in citrus fruit are significantly correlated with fruit sugar content [21]. In highly weathered soils the exchangeable Mg concentration is generally low or at deficient level [22]. Soil or foliar application of Mg fertilizers is recommended to ameliorate Mg deficiency in the field [19]. Addition of MgSO_4_ in Uganda significantly increased foliar Mg concentrations but did not significantly increase banana yields [23]. Soil application of Mg fertilizer significantly increased leaf Mg concentrations of Nanfeng tangerine (*Citrus reticulata* Blanco cv. Kinokuni) [24]. Magnesium deficiency significantly decreased coffee shoot dry matter and increased leaf soluble sugar content in solution culture [25]. Exogenous application of 30% Mg solution corrected Mg deficiency in tea plants under Mg deficiency conditions [26]. Whether or not this is also applicable to banana production is the focus of the present study.

Several soil management strategies have been used to improve soil quality for higher yields and quality of banana. Lime application is the most common method used to increase yield and quality of crops in acid soils including mango [27] and cassava [28]. However, lime application decreased soil exchangeable Mg^2+^ concentration [29] despite increases in soil pH and exchangeable Ca^2+^ concentrations [30,31]. In addition, banana foliar Mg concentrations decreased significantly with excessive application of lime [32]. Calcium magnesium phosphate fertilizer is alkaline, with an aqueous solution pH of 8.2–8.5. It is often used as a phosphorus source in agricultural production [33]. CMP has been shown to be effective in increasing soil pH and promoting sugarcane yields [34]. Whether or not it also benefits plant Mg nutrition remains to be investigated. Organic amendments such as organic manures, composts and biochar are also effective in ameliorating soil acidity and enhancing soil quality [35,36]. The application of organic manures increases soil pH, decreases exchangeable Al^3+^ concentration [30], and increases nutrient (Ca^2+^ and Mg^2+^) concentrations in acid soils [37]. In addition, organic amendments modify the structure of the soil microbiome [38], enhance soil quality and increase total soluble solids in apple trees [39]. How to synchronize soil quality, banana yield and quality is important in banana plantation.

China ranked fifth (0.43 million ha) in global banana cultivated area and second in yield produced (13.3 million tonnes, approximately 12% of world production) in 2016 [3]. By contrast, the average yield in China was 31.0 t ha^-1^, approximately half the world highest average yield and ranking 23^rd^ globally. Closing the yield gap and increasing banana quality are therefore the important challenges in Chinese banana production. The present experiment was conducted in Guangxi province where the cultivated area of banana accounts for 26.4% of the Chinese production area and ranked second in 2016. We conducted a field experiment for two years to compare lime, calcium magnesium phosphate fertilizer (CMP), an organic fertilizer (OF) and combined application of organic fertilizer and CMP on banana growth, yield and quality in a highly weathered Udic Ferralsol in south China. In addition, we investigated the effectiveness of exogenous application of MgSO_4_ solution through banana corms on the amelioration Mg deficiency and on banana quality at the fruit development stage. We hypothesized that (1) appropriate management practices can improve soil fertility and increase banana yields, (2) application of organic fertilizer is an effective strategy to improve banana quality in addition to increasing yield, and (3) short-term supply of Mg solution during the fruit development stage may increase the quality of banana.

## Material and methods

### Experimental site

The study was carried out in Long′an county, Guangxi province, south China (23.05° N, 107.82°E). The region has a typical subtropical humid monsoon climate and an annual average temperature of 21.8°C. The soil is a highly weathered Udic Ferralsol. The soil has a pH of 3.7 and with organic carbon content (SOC) of 17.3 g kg^-1^. Soil available N, available P (Olsen-P), available K, and exchangeable Ca^2+^ and Mg^2+^ concentrations were 48.3, 21.7, 46.0, 412.4 and 16.7 mg kg^-1^, respectively. Soil Fe, Mn, Cu and Zn concentrations were 18.6, 11.5, 1.13 and 10.6 mg kg^-1^, respectively. The soil Al^3+^ concentration was 608.5 mg kg^-1^. During the experimental period the precipitation was 1035 mm in 2016–2017 and 1250 mm in 2017–2018.

### Experimental design and field management

The field experiment was conducted from September 2016 to August 2018. The field was previously used to grow sugarcane and the sugarcane residues were completely cleared and the field ploughed before planting banana. A complete randomized block with three replicates of each treatment was set up. The experiment consisted of five treatments, namely a control with no external inputs (CK), lime (Lime), calcium magnesium phosphate fertilizer (CMP), organic fertilizer (OF), and organic fertilizer with calcium magnesium phosphate fertilizer (OFC). The application rates of lime and calcium magnesium phosphate fertilizer were 2.66 and 3.28 t ha^-1^, respectively. In the OF treatment the organic fertilizer application rate was 18.75 t ha^-1^. In the OFC treatment the organic fertilizer and calcium magnesium phosphate fertilizer application rates were 16.41 and 2.34 t ha^-1^, respectively. The lime was purchased from the local market and the calcium magnesium phosphate fertilizer was produced by Yunnan Kunyang Phosphate Fertilizer Factory Co., Ltd., China. The organic fertilizer was made from a mixture of filter mud from a sugar factory, plant residues (cassava residues, ground tobacco, and mushroom compost) and concentrated molasses solution (3:5:2, w/w/w) by Guangxi Jinsui Ecological Technology Co., Ltd., China. The three materials were mixed and further fermented to produce the organic fertilizer. The organic fertilizer had an organic carbon content of 30% with 1.07% N, 1.24% P, and 0.92% K. The lime, calcium magnesium phosphate fertilizer and organic fertilizer were mixed thoroughly with the surface soil (0–20 cm) before banana planting. The overall quantity of nutrients across the two growing seasons was as follows based on our previous research [40]. The uptake of N, P, K, Ca and Mg were 265–459, 37–54, 899–1493, 78–109 and 31–58 kg ha^-1^ respectively. In the first year the nutrient application rates were 501.0 kg N, 165.3 kg P (CMP, 590.4 kg; OF, 232.5 kg; OFC, 203.5 kg), 1554.2 kg K, 243.4 kg Ca and 55.1 kg Mg ha^-1^, respectively. In the second year the application rates were 474.2 kg N, 255.5 kg P (CMP, 0 kg), 1512.0 kg K, 297.9 kg Ca and 45.0 kg Mg ha^-1^. The chemical fertilizers were fertigated about 55 times (5- to 7-day intervals) in both years during the experimental period. There were five drippers per banana plant and the water dropper interval was 50 cm. Pesticides and fungicides or bactericides were sprayed monthly on the leaves to control pests and diseases.

*Musa* AAA Cavendish cv. Williams B6 was cultivated, one of the most widely planted cultivars in China. The banana seedlings were produced by tissue culture and provided by Guangxi Xiangfeng Seed Company Co., Ltd., China. The germ-free banana seedlings were firstly grown in nursery cups until the expansion of the 7^th^ leaf and then transplanted into the field. The plot size was 156 m^2^ (10.4 m × 15 m) with 30 plants at a spacing of 2 m × 2.6 m, resulting in a density of 1875 plants ha^-1^. The banana seedlings (mother plant, 1^st^ cycle) were planted in September 2016 and harvested in August 2017. After harvest uniform suckers (first ratoon) were selected for the 2^nd^ cycle and the bananas were harvested in July 2018.

### Exogenous application of Mg solution

At the fruit development stage the control plots were split into two sub-plots each 78 m^2^ (10.4 m × 7.5 m). Banana seedlings in one sub-plot were exogenously supplied with 1 mmol L^-1^ MgSO_4_ solution (CK+Mg) through the corms (S1 Fig). The application rate of MgSO_4_ solution was 200 mL on each occasion with two-day intervals over a total period of 30 days. The plants in the other sub-plots were supplied with the same amount of water as a control (CK-Mg). The plants were grown for another 13 days, and the fruits were harvested for the determination of total soluble solids and soluble sugars.

### Soil sampling and analysis

Six soil core samples were collected at depths 0–20 and 20–40 cm from banana rows at 50-cm intervals and then thoroughly mixed to give one composite sample with three replicates per treatment (S2 Fig). Soil samples were taken 106, 282 and 673 days after planting (DAP). The soil was air-dried at room temperature and then passed through a 2-mm sieve for chemical analysis. Soil pH was determined using 1 mol L^-1^ KCl suspension (1:5 soil:solution, v/v). Soil nutrients were determined according to Bao [41]. Soil organic matter content (SOM) was determined using the 0.8 mol L^-1^ K_2_Cr_2_O_7_ oxidation-reduction titration method. Available nitrogen (AN) was determined by alkaline hydrolysis and diffusion. Soil mineral nitrogen (NO_3_^—^N and NH_4_^+^-N) was extracted from fresh soil with 2 mol L^-1^ KCl (1:20, soil:water, w/v) by shaking for 1 h at 25°C and the extracts were analyzed using continuous flow analysis (TRACS 2000 system, Bran and Luebbe, Norderstedt, Germany). Available phosphorus (Olsen P) was assayed spectrophotometrically. Available K^+^ and exchangeable Ca^2+^ and Mg^2+^ were determined by flame photometry and atomic absorption spectrometry, respectively, after extraction with 1 mol L^-1^ NH_4_OAc. Exchangeable Fe^2+^, Mn^2+^ and Zn^2+^ were extracted with 0.1 mol L^−1^ HCl and determined by inductively coupled plasma-optical emission spectroscopy (ICP-OES, Optima 3300 DV, Perkin-Elmer, Waltham, MA) [42]. Exchangeable aluminum was extracted with 1 mol L^-1^ KCl and determined by an acid-alkali neutralization titrimetric method.

### Root sample collection and measurement of root length

Root samples were used to assess root distribution and were collected 106 and 282 days after planting with three replicates per treatment as shown in S2 Fig. Soil cores with 8 cm diameter × 20 cm depth were collected from plant rows and separated into two depths (0–20 and 20–40 cm). All visible roots were picked out and then washed out of the soil with tap water. The cleaned root samples from different cores were scanned using an Expression 1600 Pro Model EU-35 scanner (Epson, Suwa, Japan). The images were analyzed with the Win-Rhizo software package (Regent Instruments Inc., Quebec, Canada) to obtain total root lengths.

### Plant sampling and analysis

Shoots were sampled 106, 282, 346 and 673 days after planting with three replicates per treatment. Banana plants were divided into pseudostem and leaves 106 and 282 days after planting and the pseudostem, leaves, peduncle and fruit fingers were collected separately 346 and 673 days after planting. The fresh weights of each part were recorded in the field and sub-samples were collected according to Nyombi et al. [43]. The sub-samples of pseudostem, peduncle and fruit fingers were collected from the upper, middle and lower parts. Leaf sub-samples were collected from the middle part of each fully expanded leaf. Fruit finger sub-samples were divided into peel and pulp. All subsamples were weighed, chopped, oven-dried at 105°C for 30 min and then at 70°C to a constant weight. The sub-samples were then ground using a stainless steel mill for nutrient determination. Fresh fruit fingers were ripened using 0.7–1.0‰ ethephon solution at 20°C for determination of total soluble solids and soluble sugars.

Samples (pseudostems, leaves, peduncles, peels and pulps) were digested in a mixture of concentrated HNO_3_ and H_2_O_2_ in a microwave-accelerated reaction system (CEM, Matthews, NC). The digests were analyzed for P, K, Ca, Mg, Fe, Mn, Cu, Zn and Al by ICP-OES. Total soluble solids were determined using the pulps. The pulps were from the middle section of the fruit fingers. They were beaten into a puree which was placed in a refractometer [44]. The soluble sugar content was determined by the anthrone-sulfuric acid method [45].

### Statistical analysis

Normality and homogeneity of variance of all data were checked before analysis of variance. Univariate analysis of variance was used for soil physico-chemical properties, shoot biomass, nutrient concentrations in leaves, nutrient contents, total soluble solids content and soluble sugar content with the SPSS16.0 for Windows (SPSS Institute, Inc., Cary, NC) software package. Differences between treatments according to Duncan's multiple range test at the 5% level were regarded as statistically significant. Comparisons of total soluble solids content and soluble sugar content between CK-Mg and CK+Mg treatments were conducted by independent-sample T-tests (two-tailed) at *P*<0.05. Regression analysis was used to describe the relationships between yield, leaf nutrient concentrations and shoot biomass, and between leaf Mg concentration and total soluble solid content. The spatial distribution of root length was obtained using Surfer 10.0 (Golden Software, Golden, CO).

## Results

### Shoot biomass and banana yield

**Fig 1:**
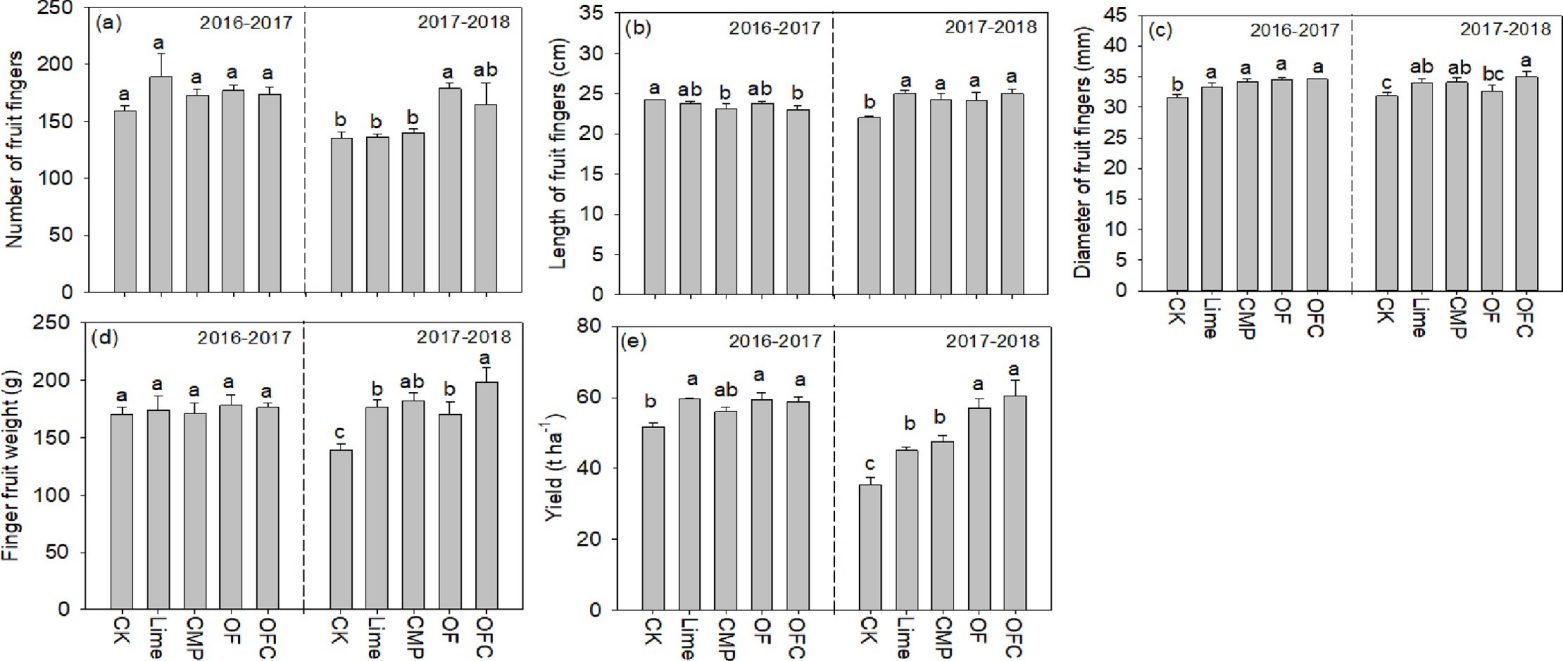
Banana yields and yield components in 2016–2017 and 2017–2018. Plants were grown in soil amended with lime (Lime), calcium magnesium phosphate fertilizer (CMP), organic fertilizer (OF), organic fertilizer with calcium magnesium phosphate fertilizer (OFC) or remained unamended (CK). Values are means ± SE (n = 3). Different lowercase letters within each column denote significant differences among different fertilization treatments in 2016–2017 and 2017–2018 (*P* < 0.05).

**Fig 2:**
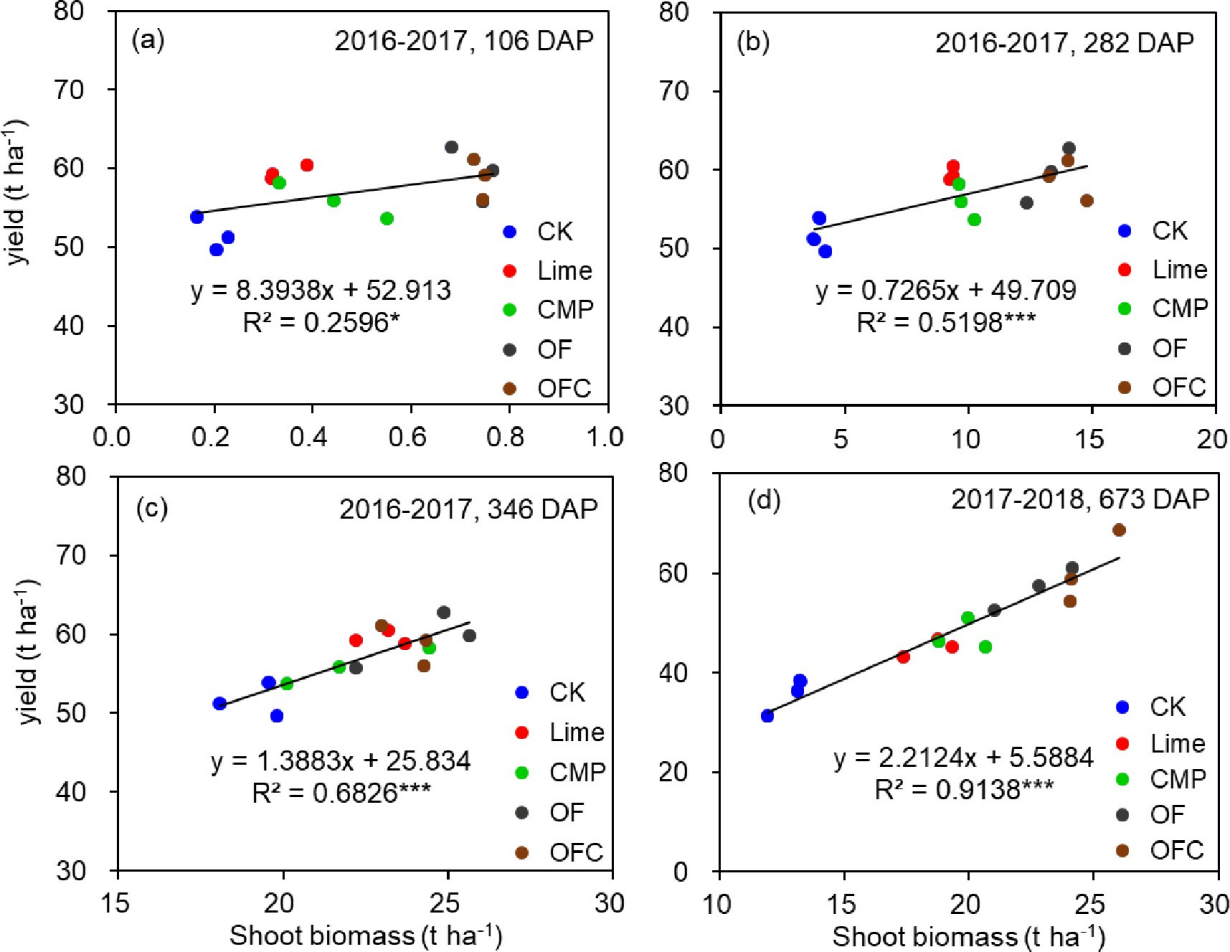
Relationship between yield and shoot biomass of banana in 2016–2017 and 2017–2018. The plants were grown in soil amended with lime (Lime), calcium magnesium phosphate fertilizer (CMP), organic fertilizer (OF), organic fertilizer with calcium magnesium phosphate fertilizer (OFC) or remained unamended (control, CK). Asterisks indicate significant differences at *P* < 0.05 (*) or *P*< 0.001 (***).

Soil amendments significantly increased shoot biomass and yields of banana compared to the control in both 2016–2017 and 2017–2018 (Fig 1, S3 Fig). In 2016–2017 the yield increase (8.3–15.3%) in the soil amendments treatment was due mainly to the significantly increased diameter of fruit fingers and possibly to the number of fruit fingers. No significant difference was observed among different soil amendments treatments and CMP tended to give the lowest yield among the soil amendments treatments. In 2017–2018 the banana yields in the OF (57.0 t ha^-1^) and OFC (60.6 t ha^-1^) treatments were significantly higher than in the Lime and CMP treatments. The yields in the latter two were significantly higher than in the control, with increases of 27.5 and 34.5%, respectively. The yield increases in OF and OFC were due to significant increases in the number of fruit fingers (18.4–32.5% increase), while those in the Lime and CMP treatments were due to the diameters of the fruit fingers and the finger fruit weights. The lengths of fruit fingers in the soil amendments treatments were significantly higher than in the control. Shoot biomass in the control was significantly lower than in the soil amendments treatments (S3 Fig). Shoot biomass in the Lime and CMP treatments was significantly lower than in the OF and OFC treatments 102, 282 and 673 days after planting. After 346 days the shoot biomass was significantly higher in the Lime, OF and OFC treatments than in the control, and no significant difference was observed among the soil amendments treatments (S3 Fig). There were significant positive correlations between shoot biomass and banana yields across all sampling times (Fig 2), and the correlation coefficients increased with increasing plant growing period. By 106 and 282 days after planting, compared to the control and the Lime treatment, root lengths in the CMP, OF and OFC treatments were higher and the roots had extended deeper down the soil profile (S4 Fig).

### Foliar nutrient uptake

**Fig 3:**
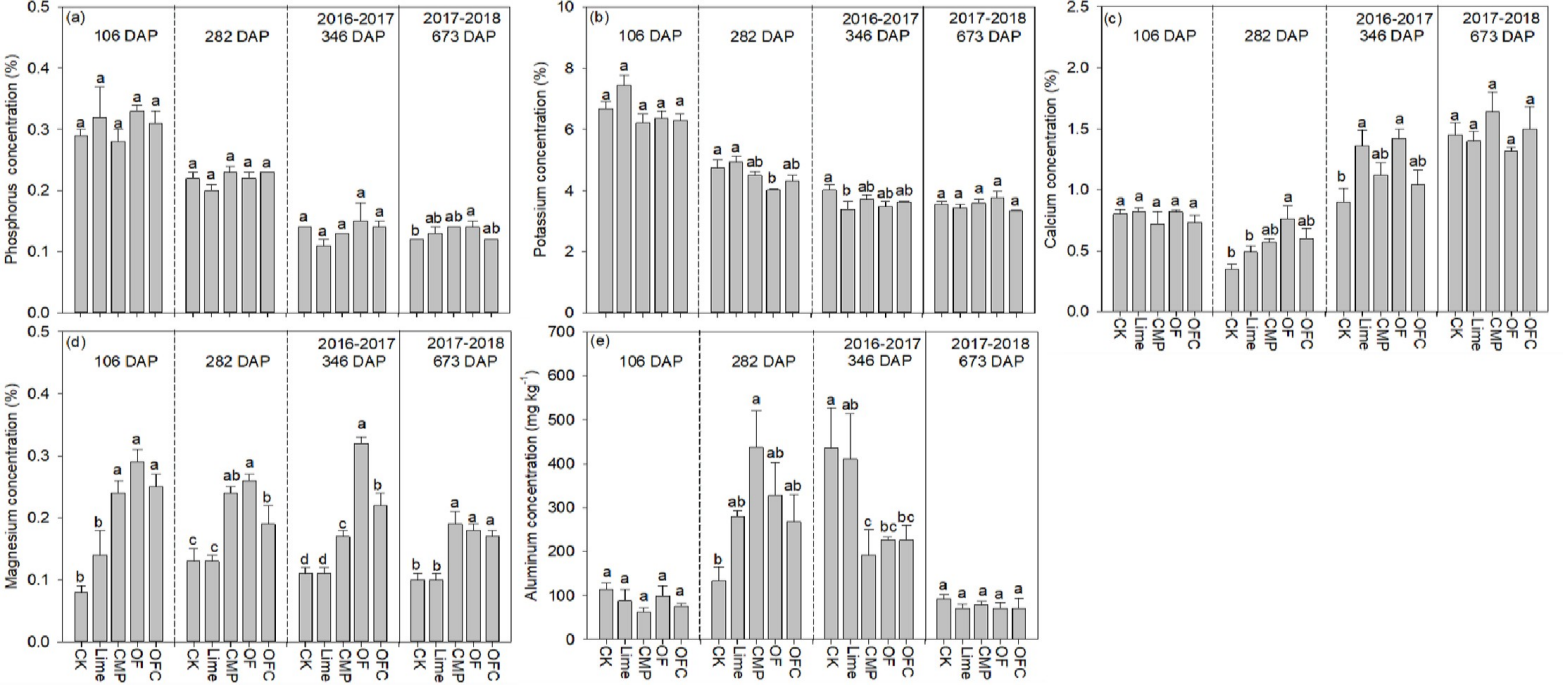
Effect of different soil amendments treatments on nutrient concentrations in banana leaves. The plants were grown in soil amended with lime (Lime), calcium magnesium phosphate fertilizer (CMP), organic fertilizer (OF), organic fertilizer with calcium magnesium phosphate fertilizer (OFC) or remained unamended (CK). Values are means ± SE (n = 3). Different lowercase letters within each column denote significant differences among different treatments in 2016–2017 and 2017–2018 (*P*<0.05).

Leaf Mg concentrations in the CMP, OF and OFC treatments were consistently higher (1.5–3.6 times) than in the control and the Lime treatment across all sampling times (Fig 3). No significant difference was observed among the three soil amendments treatments 106 and 673 days after planting. Leaf K concentrations were lowest in the OF (282 days) and Lime (346 days) treatments, and the highest values were in the Lime (282 days) treatment or the control (346 days). No significant difference was observed among different treatments at 106 or 673 days after planting. Similarly, in general, leaf Ca concentrations increased in the Lime (except at 282 days) and OF treatments relative to the control 282 and 346 days after planting, and no significant difference was observed among the soil amendments treatments 106 and 673 days after planting. Leaf Al concentrations in the control were significantly higher than in the CMP, OF and OFC treatments 346 days after planting. However, no significant difference was observed at other harvests, except higher leaf Al concentrations in the CMP treatment relative to the control after 282 days. In general, foliar P concentrations did not differ significantly among treatments across all sampling times, except that P concentrations in the control were significantly lower than in OF after 673 days (Fig 3). Leaf Fe, Cu and Zn concentrations didn’t show constant trends. But Mn concentration tended to decrease in the amended treatments except at 282 days (S1 Table). Leaf Mg concentration was significantly positively correlated with shoot biomass cross all sampling times, whilst significant correlations between leaf K or Ca concentration with shoot biomass were only observed at 282 days (Table 1). Leaf Mn concentration was significantly negatively correlated with shoot biomass at 106 and 673 days. Leaf P, Fe, Cu and Zn concentrations were not significantly correlated with shoot biomass at either sampling time (S2 Table).

**Table 1:** Regression analysis between leaf nutrient concentration and shoot biomass.

Year	Days after planting	Leaf nutrient	Regression equation	R^2^	*P*
2016–2017	106	K	y = -0.140x + 1.415	0.136	0.177
		Ca	y = -0.370x + 0.780	0.023	0.592
		Mg	y = 1.974x + 0.098	0.577	**0.001**
	282	K	y = -4.859x + 31.961	0.307	**0.032**
		Ca	y = 16.098x + 1.194	0.549	**0.002**
		Mg	y = 38.195x + 2.824	0.367	**0.017**
	346	K	y = -3.435x + 35.009	0.263	0.051
		Ca	y = 4.375x + 17.376	0.262	0.051
		Mg	y = 16.579x + 19.395	0.368	**0.017**
2017–2018	673	K	y = -0.542x + 21.587	0.001	0.916
		Ca	y = -0.892x + 20.974	0.002	0.878
		Mg	y = 68.238x + 9.707	0.461	**0.005**

Magnesium contents in the OF and OFC treatments were significantly higher than in the control (2.63–12.9 times) and the Lime (1.82–4.40 times) treatment at all harvests. No significant difference was observed between the OF and OFC treatments after 106, 346 and 673 days. In general, the P, K and Ca contents in the control were significantly lower than in the soil amendments treatments at all sampling times. The P and K contents in the OF and OFC treatment were significantly higher than in the Lime and CMP treatments after 106 and 282 days, except K in OF treatments 282 days after planting. The Al contents in the CMP, OF and OFC treatments did not differ significantly across all sampling times, except that Al content in the OFC treatment was significantly higher than in the control after 282 and 673 days. The contents of Fe, Cu, Mn and Zn in the OF and OFC treatments were generally significantly higher or showed an increasing trend compared to other treatments at all sampling times (S3 Table).

### Total soluble solids and soluble sugar contents

**Fig 4:**
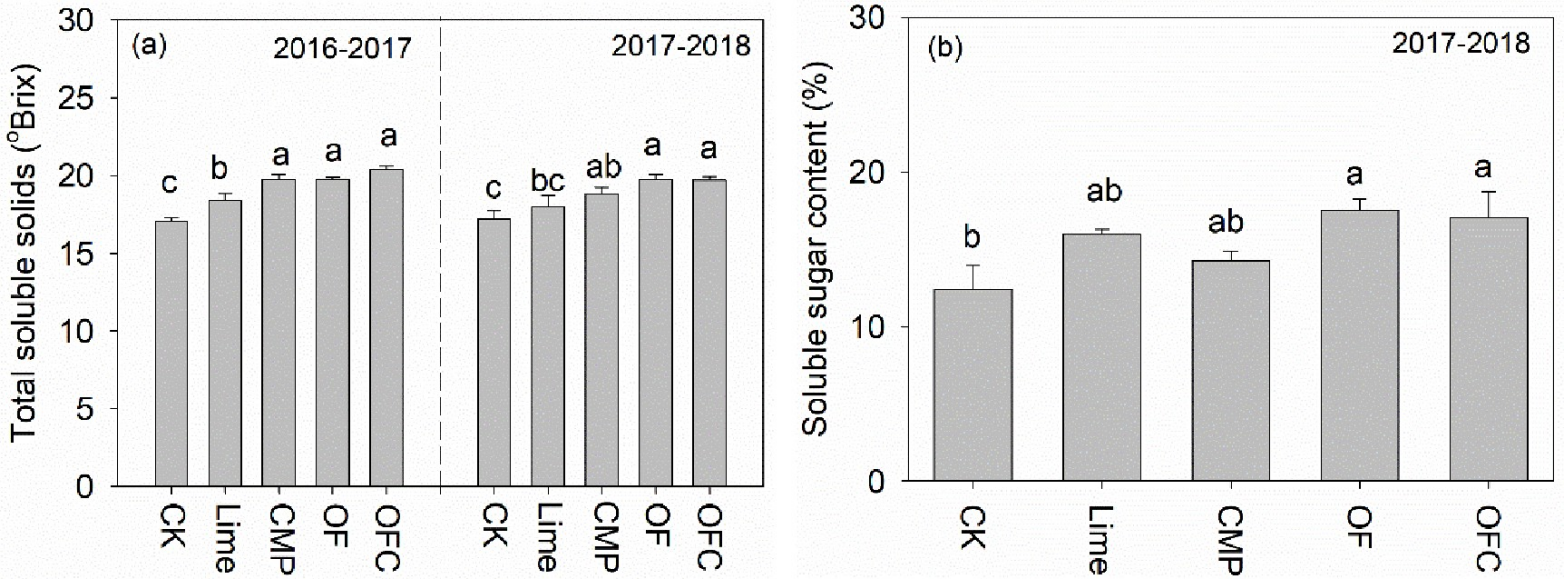
(a) Total soluble solids and (b) soluble sugar content of banana fruits in different years. The plants were grown in soil amended with lime (Lime), calcium magnesium phosphate fertilizer (CMP), organic fertilizer (OF), organic fertilizer with calcium magnesium phosphate fertilizer (OFC) or remained unamended (control, CK). Values are means ± SE (n = 3). Different lowercase letters above the bars denote significant differences among treatments in 2016–2017 and 2017–2018 (*P* < 0.05).

**Fig 5:**
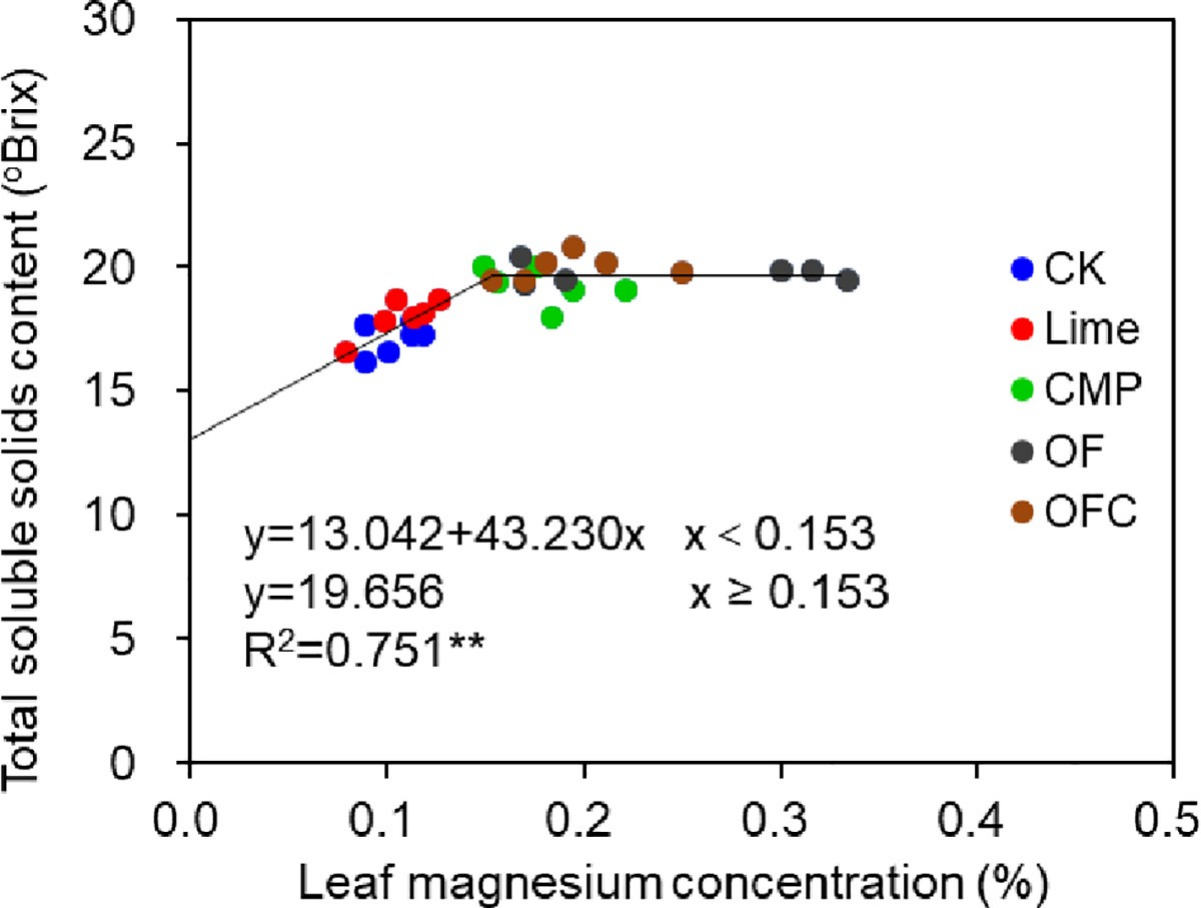
Relationship between leaf Mg concentration and total soluble solids content. The plants were grown in soil amended with lime (Lime), calcium magnesium phosphate fertilizer (CMP), organic fertilizer (OF), organic fertilizer with calcium magnesium phosphate fertilizer (OFC) or remained unamended (control, CK). Asterisks indicate significant difference at *P* < 0.01(**).

Compared to the control, total soluble solids and soluble sugar contents in banana pulp were significantly higher in the soil amendments treatments across both years (Fig 4A). Comparing the different soil amendments treatments, total soluble solids were lowest in the Lime treatment, and no significant difference was observed among the CMP, OF and OFC treatments. Compared to the control, soluble sugar contents were higher in the OF (41.3% increase) and OFC (37.5% increase) treatments followed by the Lime and CMP treatments (Fig 4B). The best-fit linear-with-plateau model predicted the relationship between leaf Mg concentration and total soluble solids content, and the critical Mg concentration was ~ 0.15%. Magnesium concentrations in the control and in Lime were below this level whilst the values in the organic fertilizer and CMP treatments were above (Fig 5).

### Soil physico-chemical properties

**Fig 6:**
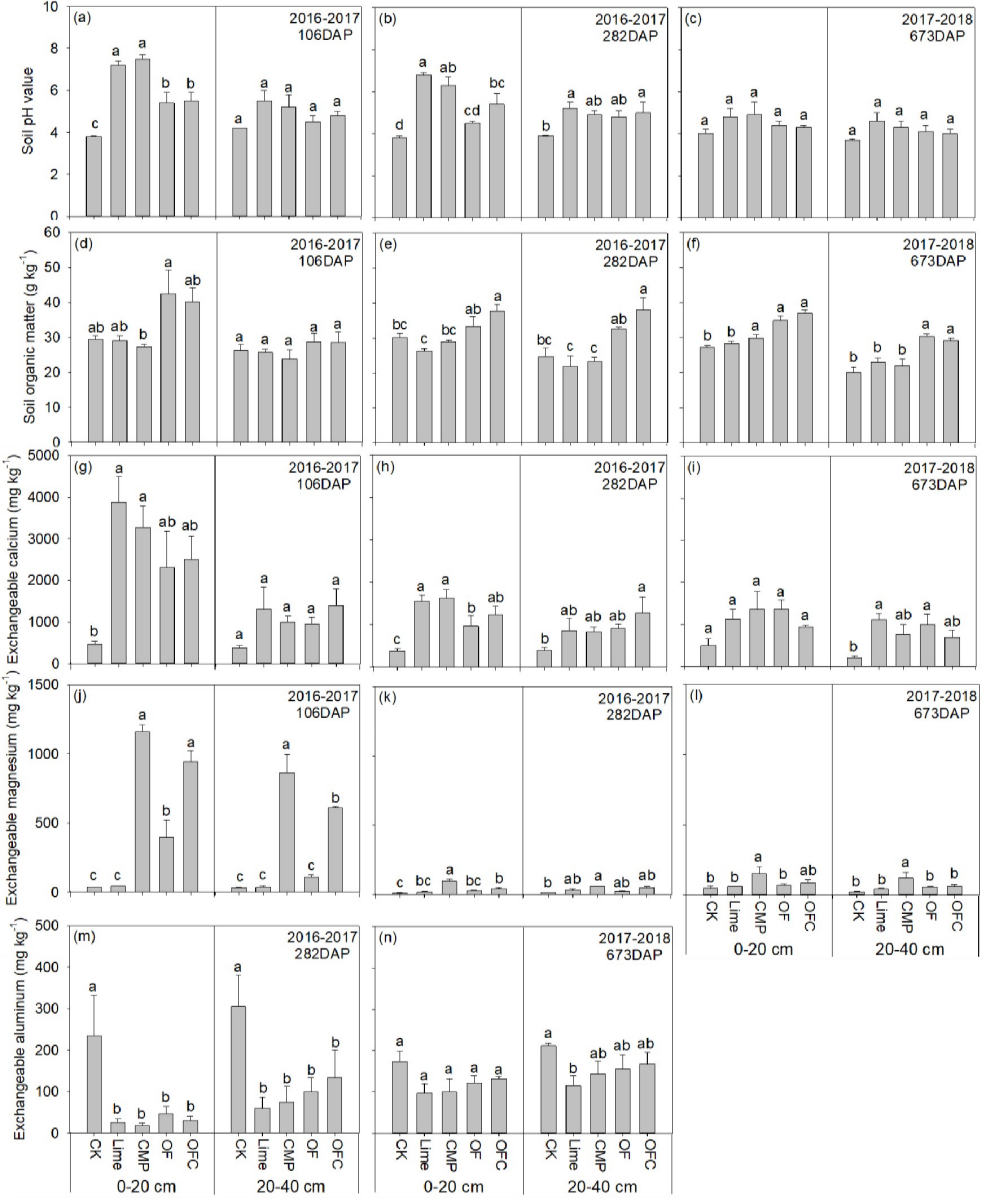
Effect of different soil amendments treatments on selected soil physico-chemical properties. The soil was amended with lime (Lime), calcium magnesium phosphate fertilizer (CMP), organic fertilizer (OF), organic fertilizer with calcium magnesium phosphate fertilizer (OFC) or remained unamended (CK). Values are means ± SE (n = 3). Different lowercase letters within each column denote significant differences among different treatments in 2016–2017 and 2017–2018 (*P*<0.05). SOM, soil organic matter.

In the top 20 cm of the soil profile, soil amendments significantly increased soil pH after106 and 282 days (Fig 6). In general, the pH increase was higher in the Lime (average 6.3) and CMP (average 6.2) treatments compared to OF (average 4.8) and OFC (average 5.1) at all sampling times. No significant difference among treatments was observed at both soil depths after 673 days and at 20–40 cm depth after 106 days. At 0–20 cm and 20–40 cm depths the SOM contents in the organic fertilizer treatments (OF and OFC) were comparable or significantly higher than in the control, limed or CMP plots, while SOM contents in the Lime and CMP treatments did not differ significantly from the control at any sampling time. In general, the soil exchangeable Ca concentration was lower in the control (208–497 mg kg^-1^) than in the soil amendments treatments (691–3876 mg kg^-1^) at both soil depths across all sampling times. No significant difference among soil amendments treatments was observed except that the exchangeable Ca concentration in the OF treatment was significantly lower than in the Lime and CMP treatments at 0–20 cm depth after 282 days. In general, at all sampling times and both soil depths, soil exchangeable Mg concentrations in the control (11.8–49.1 mg kg^-1^) and the Lime (17.3–59.8 mg kg^-1^) treatment were lower than in CMP (58.2–1159 mg kg^-1^), and the highest value was in the CMP treatment followed by the OFC (41.4–945 mg kg^-1^) and OF (24.4–398 mg kg^-1^) treatments. Soil Al concentration in the control was significantly higher than in the other treatments at both soil depths 282 days after planting. No significant difference was observed among the soil amendments treatments at both soil depths after 282 and 673 days. The addition of organic fertilizer (OF and OFC treatments) significantly increased soil available N after 106 days and the NH_4_^+^-N concentration after 673 days in the top 20 cm of the soil profile. Soil available P concentration in the CMP treatment were highest and no significant differences were observed among the other treatments at all sampling times and both soil depths, except at 20–40 cm depth after 282 days after planting (S4 Table). Soil Fe concentration in the OF and OFC treatments were significantly higher than that in the Lime and CMP treatments in the 20 cm soil profile at 106 and 282 days, while no significant difference was observed at 20–40 cm depth. Soil Mn concentrations in the OF and OFC treatments were higher compared to other treatments at both depths at 282 and 673 days. At 106 days, soil Cu concentration did not differ significantly among different treatments (except in the CMP treatment) at both soil depths, while at 673 days the Cu concentration in the OF and OFC treatments was significantly higher than in the other treatments at both soil depths. Soil Zn concentration did not differ significantly among the treatments at all sampling times at both soil depths (except in 20–40 cm at 673 days) (S4 Table).

### Total soluble solids in response to exogenous application of Mg solution

**Fig 7:**
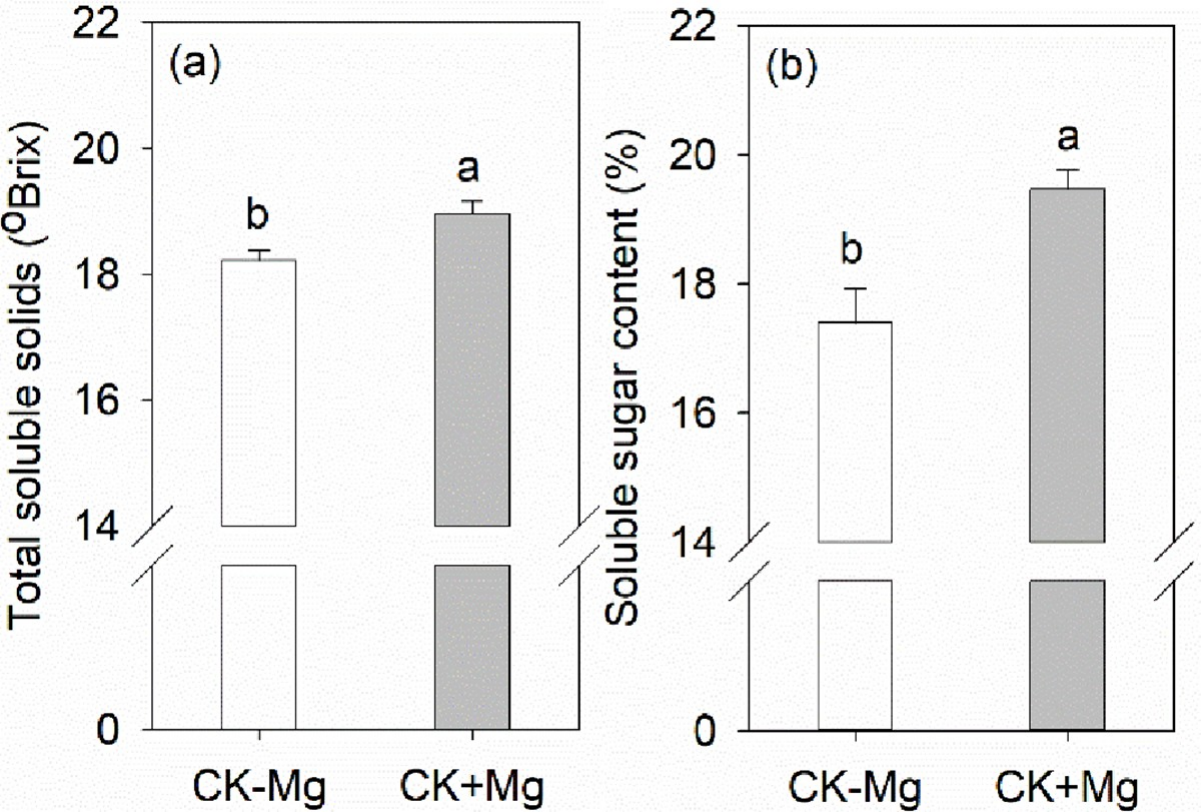
(a) Total soluble solids and (b) soluble sugar content in banana fruits. Plants grown in the control were amended with Mg solution (CK+Mg) or without Mg solution (CK-Mg) for 30 days. Values are means ± SE (n = 3). Different lowercase letters above the bars denote significant differences between CK-Mg and CK+Mg (*P*<0.05).

Application of Mg solution significantly increased total soluble solids and soluble sugar contents (Fig 7). The total soluble solids and soluble sugar contents in the CK+Mg treatment increased by 4.0 and 11.9%, respectively, relative to the CK-Mg treatment.

## Discussion

As expected, the application of lime, CMP and organic fertilizers increased soil pH, SOM (only in OF and OFC treatments) and the availability of soil nutrients (K, Ca, Mg) and decreased soil Al concentrations (Fig 6, S4 Table). Consequently, shoot growth and yields of banana increased significantly in the soil amendments treatments, with the highest values occurring in the OF and OFC treatments. Our results provide strong evidence that soil amendments are important practices in increasing banana yields in subtropical acid soils. Acid soils are constrained by low soil fertility and high soil acidity [46]. Our results show that lime, CMP and organic fertilizer are effective amendments, and this is consistent with other studies on banana [32,47] and other crops in tropical and subtropical regions [48,49,50]. Banana yields in the Lime and CMP treatments also increased significantly over the two-year period of the experiment (Fig 1). It is interesting to find that banana yields in the Lime and CMP treatments were significantly lower than in the organic fertilizer (OF) alone or in combination with CMP in the second year. The difference of yield between organic fertilizer and lime or CMP was not due to soil nutrients (N, P and K) and pH, but it was mostly due to an increase in SOM in the top soil layer and also at 20–40 cm depth at later banana growth stages (Fig 6, S4 Table). As the soil properties in the OF treatment were comparable to, or even lower than those in the CMP and Lime treatments (Fig 6, S4 Table). The importance of SOM in sustaining soil fertility [51,52] is well acknowledged and our results again confirm its important role in ensuring long-term soil fertility and plant growth, which was reported in banana plantations in Hainan, China [53] and Karnataka, India [54], and also in sugarcane production [55] in subtropical regions. Furthermore, the beneficial effects of organic fertilizers are associated with improved soil structure [56], higher soil enzyme activities [57], greater root growth [58], and increased soil microbial abundance or beneficial microbes (such as *Pseudomonas*, *Bacillus*, and *Streptomyces*) [59,60]. Banana roots in the OF and OFC treatments were larger and extended deeper down the soil profile than in the other treatments (S4 Fig) with consequent increases in nutrient uptake as reflected by the higher nutrient contents in the shoots throughout the growth period (S3 Table). In the present study the yield increase in the OFC treatment was mainly attributable to the increased diameter of fruit fingers and finger fruit weights (Fig 1), while that in the OF treatment was mainly associated with the number of fruit fingers. The number of fruit fingers, banana finger lengths, diameters of fruit fingers and finger fruit weights are the important classification standards that affect banana yield and fresh fruit consumption in international trade in banana [17]. The underlying mechanisms associated with the alteration of yield components of banana plants as a result of different soil amendments deserve further investigation.

Low soil pH is the major growth constraint on acid soils. Soil amendments in the present study were effective in modifying pH (Fig 6), and this is consistent with other studies on banana and other crops [27,32,57]. Compared to organic fertilizers, Lime and CMP were more effective in increasing soil pH during the first year, with the positive effect diminishing in the second year (Fig 6). The positive effects of liming are well recorded on acid soils [30,31,61]. The alkaline nature of CMP indicates that it has the potential to modify soil pH in addition to supplying nutrients including Ca and Mg (Fig 6). Change in soil pH in the Lime and CMP treatments can be ascribed mostly to the neutralization capacity of Lime or CMP. Ca^2+^ or Mg^2+^ in the Lime and CMP treatments can induce the release of H^+^/Al^3+^ from the surfaces of soil particles and consequently the neutralization/precipitation of H^+^/Al^3+^ with alkaline ions in the soil solution. However, reacidification might occur after the depletion of alkaline ions and Ca/Mg uptake by banana [62]. In addition, soil buffering capacity is low due to low exchangeable base cation concentrations in acid soils [63]. Soil pH is dependent on the production and consumption of H^+^ in the soil [64]. During the growth of banana plants, the uptake of cations (Ca, Mg and K) and in particular nitrate nitrogen by plants may offset the effect of H^+^ consumption [65]. This may explain the low soil pH values in the second year in the current study.

The stabilizing effect of organic fertilizers on soil pH increase may be associated with the pHBC (soil pH buffering capacity) as a result of increasing SOM content. A strong positive linear relationship between pHBC and SOM has been observed in acid soils [66,67,68]. The protonation of organic anions from the dissociation of weakly acidic functional groups on soil organic matter to form neutral molecules was suggested to be the main mechanism responsible for an increase in pHBC and soil resistance to acidification induced by manure application [69]. We did not observe synergistic effects of organic fertilizer in combination with CMP (OFC treatment), and the modification of soil pH and soil cation nutrients was at a similar magnitude to that in the OF treatment (Fig 6). Taken together, in order to sustain long-term banana production, the frequency of application of soil amendments requires investigation in future studies.

The mobility of Mg is strong and Mg^2+^ is prone to leaching in the soil profile, especially in acid soils and under heavy precipitation [70]. In the present study we found that Mg nutrition is important for banana growth, yield and quality. Although the Ca concentration also increased in the soil amendment treatments, the effects were variable among different treatments and were not consistent over harvesting times. By contrast, soil, leaf Mg concentrations in the control and the Lime treatment were significantly lower than in other treatments (Fig 3), and mostly importantly, the effect was consistent across the banana growth period. In addition, leaf Mg concentration was significantly and positively correlated with shoot growth (Table 1). Magnesium is the central atom of chlorophyll [71] and Mg deficiency results in lower chlorophyll contents and thus affects plant growth [72]. In our experiment, leaf SPAD values in the control (34.7) and the Lime (41.6) treatments were significantly lower than in the CMP (49.8), OF (51.5) and OFC (52.7) treatments. Banana plants showed Mg deficiency in the control and the Lime treatment according to nutrient diagnostic criteria [17], although no visible Mg deficiency symptoms were observed (Fig 3). Low soil Mg concentrations in acid soils [46] and the antagonistic effect of Mg^2+^ uptake with H^+^ in black pepper under nutrient solution conditions [73] have been reported. In our experiment, soil pH had a significant positive correlation with soil Mg concentration, indicating the importance of increasing pH in the amelioration of Mg deficiency. In addition to pH, Al stress [74] or K^+^ [75] may reduce leaf Mg concentrations. Low foliar Mg concentrations in the Lime treatment may be attributable to lime-induced antagonistic effects on Mg uptake [76]. For example, gypsum application caused Mg^2+^ leaching to deeper parts of the soil profile [77]. Similarly, heavy application of lime significantly decreased leaf Mg concentrations in banana fields [32]. The antagonistic effect of Mg^2+^ with other cations in banana plants deserves further investigation in banana production.

Magnesium affects photosynthesis and carbohydrate partitioning [20,78], and plays a crucial role in loading and exporting carbohydrates by the phloem from source to sink organs [79,80]. The increase in total soluble solids and soluble sugar content observed in the CMP and OF/OFC treatments (Fig 4) may be related to Mg-mediated carbohydrate translocation. Indeed, we found that leaf Mg concentration was significantly and positively correlated with the total soluble solids content of banana fruits (Fig 5). The significant increase in total soluble solids and sugar content resulting from the short-term exogenous application of Mg solution (Fig 7) implies that Mg is effective in promoting banana quality and it should be incorporated in management strategies to increase banana quality. We applied Mg solution during the fruit development period when the Mg uptake of fruit accounts for more than 20% of the whole plant, and more starch grains (carbohydrates) were expected to allocate to banana fruits until fruit maturity [17]. Soil and foliar application of Mg are common management practices to improve soil and foliar Mg concentrations [19]. For example, in Nanfeng tangerine (*Citrus reticulata* Blanco cv. Kinokuni), soil application of Mg fertilizer significantly increased leaf Mg concentrations [24]. Foliar application of Mg increased the growth, yield, and essential oil yield of oregano [81]. We made exogenous application of Mg solution through the banana corms. Banana roots and fruit bunches arise from the corms which are also important storage organs and nutrient reservoirs for sustaining bunch and fruit growth [17]. Our results indicate that short-term exogenous application of Mg solution during fruit development has great potential to overcome Mg deficiency by the short-term application of Mg. In the future, the mechanism of applying organic fertilizer and Mg solution in enhancing banana quality needed to investigated. Also, appropriate management of Mg fertilizer in banana plantation also needs to be verified in the field plots.

## Conclusions

Liming and organic fertilizer are considered to be important tools for the amelioration of acids in subtropical Udic Ferralsols. The use of organic amendments as an alternative to wholly or partly replace synthetic fertilizers is recommended to increase soil fertility and crop yields. Our results highlight the importance of organic fertilizer in integrated management strategies to ensure banana productivity and quality. In addition to the commonly observed increase in soil pH and cation availability as a result of the application of lime and CMP, the application of organic fertilizer (OF and OFC treatments) significantly promoted soil organic matter content and banana yield and quality (e.g. total soluble solids and soluble sugar content). A consistent increase in Mg concentrations in banana leaves during the two years of the experiment in the CMP and organic fertilizer treatments indicates that Mg is essential for banana production and quality. Adding Mg from banana corms increased total soluble solids and soluble sugar content. Therefore, the application of organic fertilizer and CMP or combined with exogenous Mg solution, is recommended to increase soil health, increase nutrient use efficiency, and promote the yields and quality of banana in intensive plantations in subtropical regions.

## Supporting information

S1 Table Micro-element concentrations in banana leaves in 2016–2017 and 2017–2018. (DOCX)

S2 Table Regression analysis between leaf nutrient concentration and shoot biomass. (DOCX)

S3 Table Nutrient contents in banana shoots in 2016–2017 and 2017–2018. (DOCX)

S4 Table Effects of different soil amendment treatments on selected soil physico-chemical properties. (DOCX)

S1 Fig Exogenous application of Mg solution through a banana corm. (DOCX)

S2 Fig Diagrammatic representation of the banana rows and soil core sampling in the field plots. (DOCX)

S3 Fig Banana shoot biomass in (a) 2016–2017 and (b) 2017–2018.
(DOCX)

S4 Fig Vertical distribution of root length of banana plants (a) 106 and (b) 282 days after planting in 2016–2017.
(DOCX)
