# Supplementary material for: Organic fertilizer application and Mg fertilizer promote banana yield and quality in an Udic Ferralsol
Source: PLoS One. 2020 Mar 18;15(3):e0230593. doi: 10.1371/journal.pone.0230593 (PMC7080258; doi:10.1371/journal.pone.0230593)
Supplement: S1 Table — (DOCX) [file pone.0230593.s001.docx]

**S1 Table. Micro-element concentrations in banana leaves in 2016-2017 and 2017-2018.**

| Year | Days after planting | Treatment | Fe (mg kg^-1^) | Mn (%) | Cu (mg kg^-1^) | Zn (mg kg^-1^) |
| --- | --- | --- | --- | --- | --- | --- |
| 2016-2017 | 106 | CK | 134±8.4a | 0.16±0.00a | 8.23±0.11a | 20.4±4.1a |
|  |  | Lime | 126±12.0a | 0.12±0.01b | 8.30±0.60a | 16.7±1.1a |
|  |  | CMP | 91±9.4b | 0.10±0.01b | 7.04±0.68a | 14.9±3.3a |
|  |  | OF | 119±10.5ab | 0.08±0.01b | 8.68±0.65a | 19.2±3.0a |
|  |  | OFC | 104±4.3ab | 0.09±0.02b | 8.42±0.34a | 14.5±0.6a |
|  | 282 | CK | 121±7.6b | 0.09±0.01b | 8.50±0.46a | 17.9±1.0a |
|  |  | Lime | 225±7.8ab | 0.13±0.01b | 7.81±0.60a | 15.6±0.8a |
|  |  | CMP | 272±41.3a | 0.13±0.01b | 8.50±0.52a | 17.7±2.2a |
|  |  | OF | 247±42.4a | 0.21±0.01a | 9.48±1.46a | 21.2±3.1a |
|  |  | OFC | 207±35.5ab | 0.14±0.03b | 7.91±1.41a | 19.0±1.8a |
|  | 346 | CK | 289±53.8a | 0.35±0.03a | 5.30±0.23a | 19.1±0.5a |
|  |  | Lime | 278±63.5a | 0.34±0.02a | 3.89±0.32b | 13.5±1.1b |
|  |  | CMP | 159±26.1b | 0.38±0.03a | 4.36±0.27ab | 19.0±0.3b |
|  |  | OF | 150±5.8b | 0.30±0.04ab | 4.76±0.13ab | 16.8±2.0ab |
|  |  | OFC | 155±11.9b | 0.23±0.01b | 4.76±0.45ab | 16.2±1.3ab |
| 2017-2018 | 673 | CK | 133±14.4a | 0.40±0.04a | 5.66±0.12a | 48.1±5.9a |
|  |  | Lime | 102±14.7a | 0.37±0.03ab | 5.90±0.47a | 33.7±0.8ab |
|  |  | CMP | 130±16.0a | 0.32±0.03ab | 5.17±0.17a | 30.4±4.3b |
|  |  | OF | 102±12.6a | 0.30±0.01b | 5.89±0.55a | 44.7±5.0ab |
|  |  | OFC | 89±13.9a | 0.31±0.02b | 6.47±1.72a | 38.6±7.4ab |

Note: The plants were grown in soil treated with lime (Lime), calcium magnesium phosphate fertilizer (CMP), organic fertilizer (OF), organic fertilizer with calcium magnesium phosphate fertilizer (OFC) or remained unamended (control, CK). Values are means ± SE (n = 3). Different lowercase letters above the bars denote significant differences among treatments in 2016-2017 and 2017-2018 (*P*＜0.05).
