# Supplementary material for: Organic fertilizer application and Mg fertilizer promote banana yield and quality in an Udic Ferralsol
Source: PLoS One. 2020 Mar 18;15(3):e0230593. doi: 10.1371/journal.pone.0230593 (PMC7080258; doi:10.1371/journal.pone.0230593)
Supplement: S2 Table — (DOCX) [file pone.0230593.s002.docx]

**S2 Table.** **Regression analysis between leaf nutrient concentration and shoot biomass**

| Year | Days after planting | Leaf nutrient | Regression equation | R^2^ | *P* |
| --- | --- | --- | --- | --- | --- |
| 2016-2017 | 106 | P | y = 0.868x + 0.227 | 0.031 | 0.532 |
|  |  | Fe | y = -0.003x + 0.885 | 0.100 | 0.251 |
|  |  | Mn | y = -4.633x + 1.002 | 0.530 | **0.002** |
|  |  | Cu | y = 0.028x + 0.267 | 0.014 | 0.677 |
|  |  | Zn | y = -0.012x + 0.689 | 0.056 | 0.396 |
|  |  | Al | y = -0.001x + 0.582 | 0.021 | 0.605 |
|  | 282 | P | y = 57.317x - 2.682 | 0.046 | 0.444 |
|  |  | Fe | y = 0.025x + 4.757 | 0.221 | 0.077 |
|  |  | Mn | y = -35.970x + 14.757 | 0.067 | 0.352 |
|  |  | Cu | y = 0.027x + 9.855 | 0 | 0.968 |
|  |  | Zn | y = 0.379x + 3.143 | 0.124 | 0.197 |
|  |  | Al | y = 0.010x + 7.170 | 0.132 | 0.183 |
|  | 346 | P | y = -17.867x + 24.806 | 0.034 | 0.514 |
|  |  | Fe | y = -0.010x + 24.492 | 0.147 | 0.159 |
|  |  | Mn | y = -10.326x + 25.769 | 0.100 | 0.252 |
|  |  | Cu | y = -1.687x + 30.254 | 0.247 | 0.060 |
|  |  | Zn | y = -0.259x + 26.857 | 0.103 | 0.243 |
|  |  | Al | y = -0.004x + 23.766 | 0.081 | 0.303 |
| 2017-2018 | 673 | P | y = 133.834x + 2.360 | 0.252 | 0.056 |
|  |  | Fe | y = -0.078x + 28.369 | 0.246 | 0.060 |
|  |  | Mn | y = -47.213x + 35.690 | 0.380 | **0.014** |
|  |  | Cu | y = 0.575x + 16.325 | 0.030 | 0.537 |
|  |  | Zn | y = -0.122x + 24.453 | 0.083 | 0.298 |
|  |  | Al | y = -0.063x + 24.503 | 0.106 | 0.236 |
