# Supplementary material for: Organic fertilizer application and Mg fertilizer promote banana yield and quality in an Udic Ferralsol
Source: PLoS One. 2020 Mar 18;15(3):e0230593. doi: 10.1371/journal.pone.0230593 (PMC7080258; doi:10.1371/journal.pone.0230593)
Supplement: S4 Table — (DOCX) [file pone.0230593.s004.docx]

**S4 Table. Effects of different soil amendment treatments on selected soil physico-chemical properties**

| Year | Days after planting | Soil depth (cm) | Property | Control | Lime | CMP | OF | OF+CMP |
| --- | --- | --- | --- | --- | --- | --- | --- | --- |
| 2016-2017 | 106 | 0-20 cm | AN (mg kg^-1^) | 99.4±8.5b | 94.4±4.1b | 88.8±8.1b | 134±4.4a | 130±9.8a |
|  |  |  | AP (mg kg^-1^) | 70.1±7.1b | 121±39.1ab | 171±24.2a | 112±3.7ab | 88.0±10.3b |
|  |  |  | AK (mg kg^-1^) | 90.0±16.4b | 110±19.8ab | 101±20.3ab | 136±33.2ab | 165±8.8a |
|  |  |  | Fe-HCl (mg kg^-1^) | 21.0±1.5bc | 11.2±1.1c | 6.5±2.3c | 38.9±9.4a | 31.9±0.8ab |
|  |  |  | Mn-HCl (mg kg^-1^) | 14.5±2.1b | 14.1±2.5b | 46.9±2.6ab | 65.7±20.1a | 49.2±9.1ab |
|  |  |  | Cu-HCl (mg kg^-1^) | 1.2±0.05a | 0.7±0.08ab | 0.3±0.08b | 1.1±0.22a | 1.0±0.2a |
|  |  |  | Zn-HCl (mg kg^-1^) | 12.5±1.05a | 11.9±3.4a | 16.2±1.90a | 22.7±5.74a | 15.5±2.6a |
|  |  | 20-40 cm | AN (mg kg^-1^) | 85.7±18.7ab | 108±20.4ab | 63.2±12.6b | 131±27.8a | 95.1±4.5ab |
|  |  |  | AP (mg kg^-1^) | 74.6±4.2a | 89.0±17.2a | 120±34.9a | 71.6±2.5a | 69.4±5.2a |
|  |  |  | AK (mg kg^-1^) | 80.0±18.9ab | 70.0±8.8ab | 36.7±3.6b | 114±20.2a | 133±25.3a |
|  |  |  | Fe-HCl (mg kg^-1^) | 15.2±2.1a | 16.1±0.8a | 20.1±4.5a | 27.4±4.1a | 25.1±5.7a |
|  |  |  | Mn-HCl (mg kg^-1^) | 12.4±0.9a | 14.3±3.3a | 26.2±10.0a | 23.7±2.4a | 22.4±3.9a |
|  |  |  | Cu-HCl (mg kg^-1^) | 1.0±0.1a | 0.9±0.07a | 0.8±0.1a | 1.2±0.1a | 1.2±0.1a |
|  |  |  | Zn-HCl (mg kg^-1^) | 16.7±2.2a | 16.1±0.7a | 13.0±0.8a | 12.9±1.8a | 15.6±2.4a |
|  | 282 | 0-20 cm | AN (mg kg^-1^) | 123±10.2a | 94.1±2.8a | 97.4±10.5a | 104±3.4a | 121±19.9a |
|  |  |  | AP (mg kg^-1^) | 39.0±10.2b | 83.0±28.3b | 196±5.1a | 80.9±5.0b | 81.3±6.5b |
|  |  |  | AK (mg kg^-1^) | 136±17.8a | 147±15.2a | 128±36.3a | 107±4.4a | 83.3±15.9a |
|  |  |  | Fe-HCl (mg kg^-1^) | 29.5±2.6ab | 15.9±0.2bc | 8.1±4.2c | 43.9±4.4a | 31.6±7.1a |
|  |  |  | Mn-HCl (mg kg^-1^) | 55.7±6.0c | 53.3±2.5c | 96.7±2.0b | 104±22.4b | 138±6.7a |
|  |  |  | Zn-HCl (mg kg^-1^) | 44.5±37.5a | 23.3±15.1a | 19.8±9.9a | 21.2±4.4a | 22.7±17.4a |
|  |  | 20-40 cm | AN (mg kg^-1^) | 98.0±2.9ab | 85.2±4.6ab | 80.0±3.5b | 100±12.8ab | 109±4.9a |
|  |  |  | AP (mg kg^-1^) | 24.4±8.5b | 27.4±16.4b | 54.5±11.6ab | 61.5±4.6ab | 76.3±8.4a |
|  |  |  | AK (mg kg^-1^) | 128±10.9b | 251±61.7a | 117±8.8b | 163±28.5ab | 133±21.7b |
|  |  |  | Fe-HCl (mg kg^-1^) | 32.5±1.0a | 24.3±2.6a | 24.1±0.7a | 30.4±3.3a | 26.6±4.7a |
|  |  |  | Mn-HCl (mg kg^-1^) | 42.2±5.5bc | 22.5±4.3c | 69.9±7.7b | 111±11.4a | 118±23.2a |
|  |  |  | Zn-HCl (mg kg^-1^) | 10.7±5.7a | 2.5±0.9a | 3.1±1.2a | 8.6±0.9a | 6.2±2.8a |
| 2017-2018 | 673 | 0-20 cm | NO_3_^-^-N | 4.4±1.3a | 4.2±0.3a | 3.2±0.2a | 8.5±3.0a | 5.6±0.6a |
|  |  |  | NH_4_^+^-N | 2.0±0.2b | 3.2±0.5b | 3.6±0.3b | 7.7±0.4a | 6.9±1.2a |
|  |  |  | AP (mg kg^-1^) | 41.4±16.0ab | 43.2±5.8ab | 87.9±22.0a | 55.9±10.4ab | 35.6±2.6b |
|  |  |  | AK (mg kg^-1^) | 285±67.8a | 367±34.1a | 333±11.5a | 288±52.8a | 311±30.8a |
|  |  |  | Mn-HCl (mg kg^-1^) | 40.3±6.9b | 56.3±7.9b | 87.1±9.3b | 242±31.0a | 236±19.1a |
|  |  |  | Cu-HCl (mg kg^-1^) | 1.6±0.1b | 1.4±0.04b | 1.5±0.2b | 5.6±1.3a | 8.3±1.7a |
|  |  |  | Zn-HCl (mg kg^-1^) | 10.4±4.8a | 35.5±26.4a | 23.3±3.5a | 17.6±5.3a | 19.2±5.8a |
|  |  | 20-40 cm | NO_3_^-^-N | 2.2±0.7a | 3.0±1.1a | 3.1±1.0a | 4.7±1.0a | 3.1±0.5a |
|  |  |  | NH_4_^+^-N | 1.8±0.4b | 1.6±0.2b | 2.3±0.3b | 6.2±2.1a | 3.3±0.4ab |
|  |  |  | AP (mg kg^-1^) | 8.3±3.2b | 16.3±2.5b | 23.1±11.4b | 47.05±5.0a | 25.0±3.8b |
|  |  |  | AK (mg kg^-1^) | 149±23.3a | 121±13.4a | 133±5.2a | 151±47.5a | 151±3.6a |
|  |  |  | Mn-HCl (mg kg^-1^) | 17.5±1.5b | 27.0±2.8b | 45.7±4.1b | 122±32.0a | 124±22.1a |
|  |  |  | Cu-HCl (mg kg^-1^) | 1.0±0.1b | 0.9±0.04b | 1.1±0.06b | 2.5±0.4a | 2.6±0.2a |
|  |  |  | Zn-HCl (mg kg^-1^) | 1.9±0.6b | 6.9±2.4a | 5.7±0.6ab | 4.5±0.8ab | 4.5±1.2ab |

Note: The plants were grown in soil treated with lime (Lime), calcium magnesium phosphate fertilizer (CMP), organic fertilizer (OF), organic fertilizer with calcium magnesium phosphate fertilizer (OFC) or remained unamended (Control).Values are means ± SE (n = 3). Different lowercase letters within each column denote significant differences among different treatments in 2016-2017 and 2017-2018 (*P* ＜ 0.05). AN, available nitrogen; AP, available phosphorus; AK, available potassium.
