# Supplementary figures and images for: Organic fertilizer application and Mg fertilizer promote banana yield and quality in an Udic Ferralsol
Source: PLoS One. 2020 Mar 18;15(3):e0230593. doi: 10.1371/journal.pone.0230593 (PMC7080258; doi:10.1371/journal.pone.0230593)

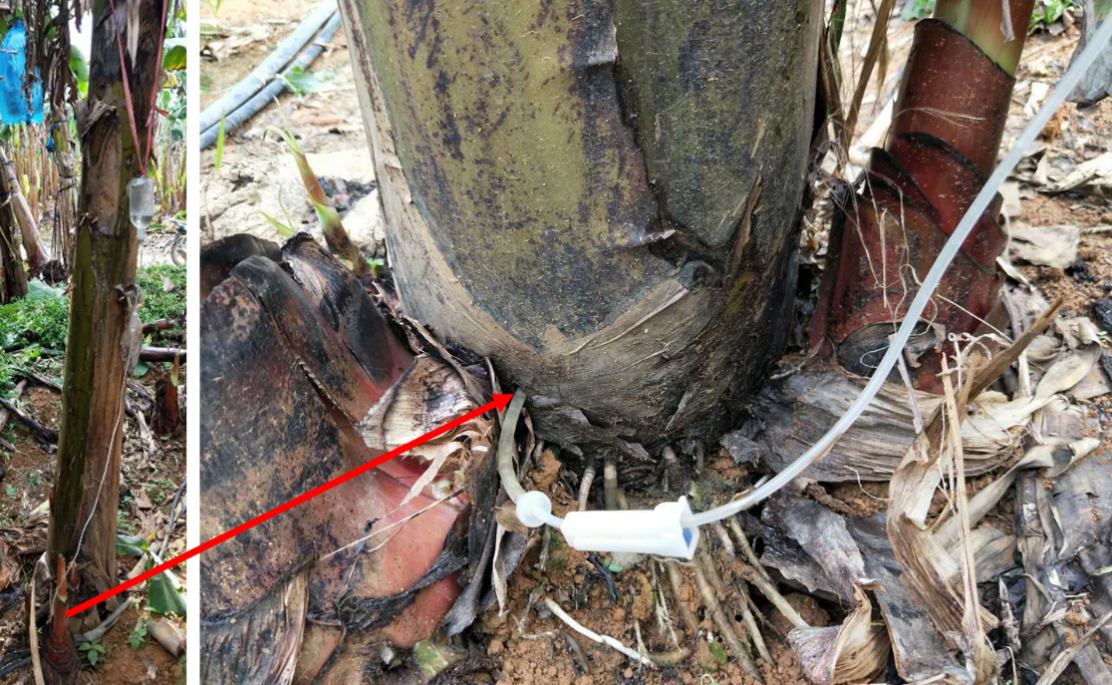


**S1 Fig.** **Exogenous application of Mg solution through a banana corm (red arrow)**

Supplement: S1 Fig — (DOCX) [file pone.0230593.s005.docx]
