# Supplementary material for: Organic fertilizer application and Mg fertilizer promote banana yield and quality in an Udic Ferralsol
Source: PLoS One. 2020 Mar 18;15(3):e0230593. doi: 10.1371/journal.pone.0230593 (PMC7080258; doi:10.1371/journal.pone.0230593)
Supplement: S2 Fig — (DOCX) [file pone.0230593.s006.docx]

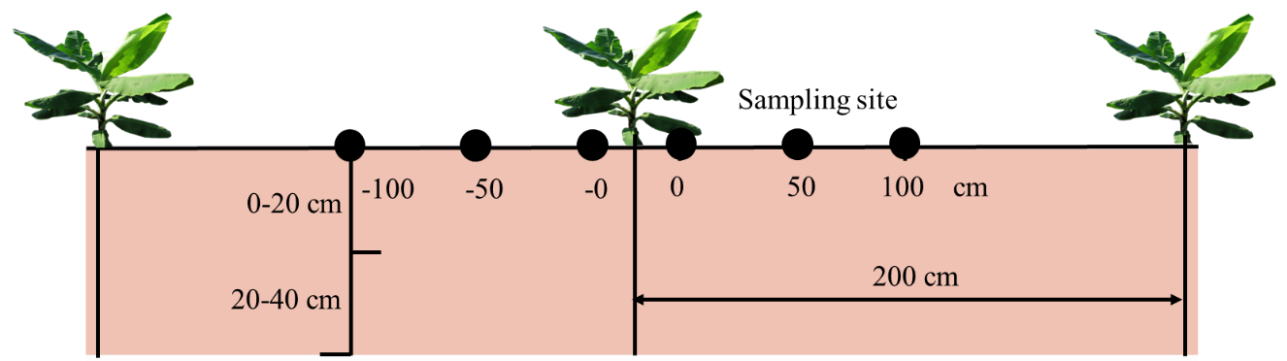


**S2 Fig.** **Diagrammatic representation of the banana rows and soil core sampling in the field plots**. Distances between rows and plants are indicated in cm. The closed circles represent the soil core sampling sites.
