# Supplementary material for: Organic fertilizer application and Mg fertilizer promote banana yield and quality in an Udic Ferralsol
Source: PLoS One. 2020 Mar 18;15(3):e0230593. doi: 10.1371/journal.pone.0230593 (PMC7080258; doi:10.1371/journal.pone.0230593)
Supplement: S4 Fig — Vertical distribution of root length of banana plants (a) 106 and (b) 282 days after planting in 2016–2017. (DOCX) [file pone.0230593.s008.docx]

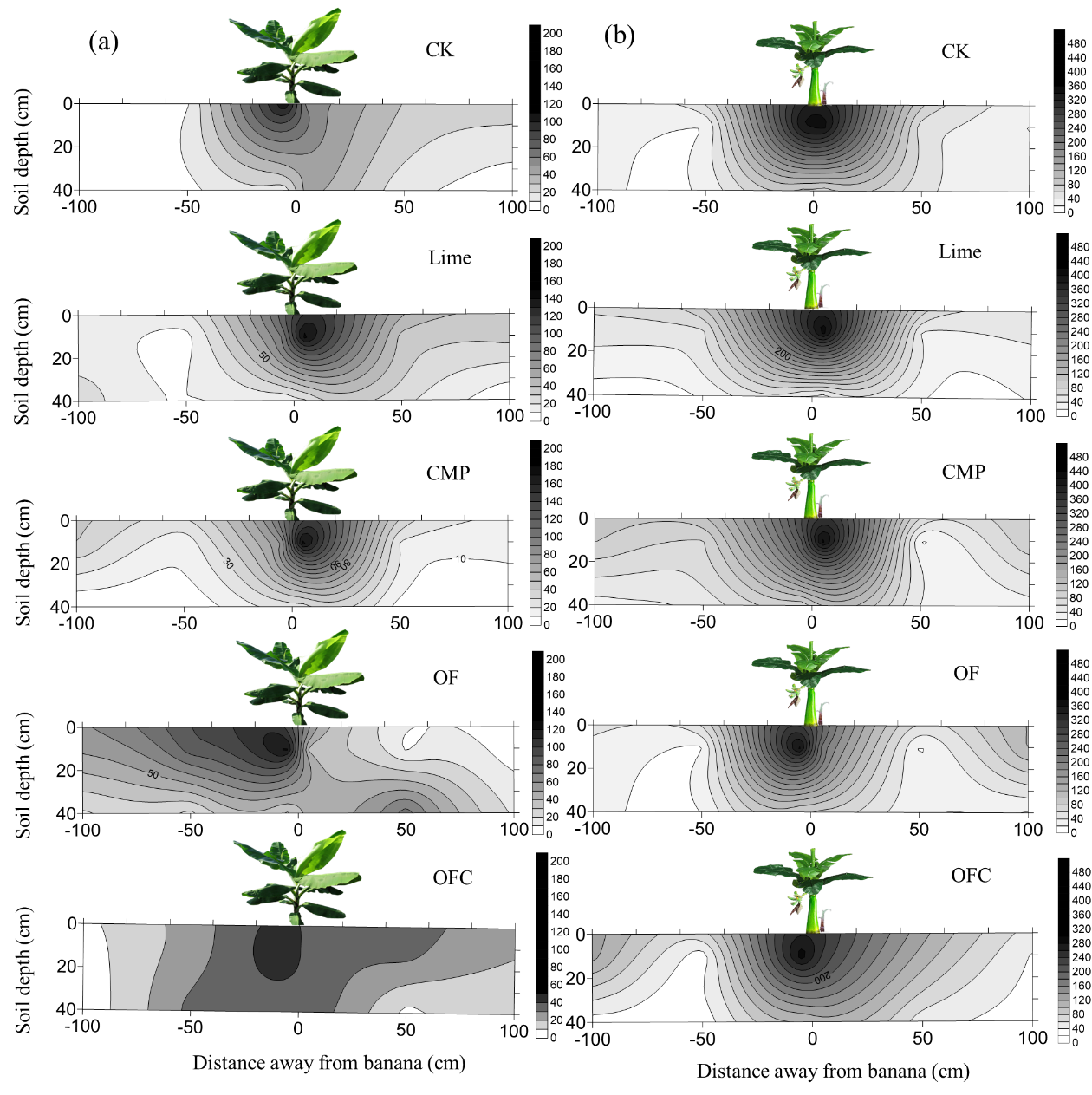


**S4 Fig.** **Vertical distribution of root length of banana plants (a) 106 and (b) 282 days after planting in 2016-2017**. Isolines represent mean values of the root length in the banana-planting row. The darker the color, the greater the root length. The plants were grown in soil treated with lime (Lime), calcium magnesium phosphate fertilizer (CMP), organic fertilizer (OF), organic fertilizer with calcium magnesium phosphate fertilizer (OFC) or unamended (control, CK).
